# Schwannome benin primitif retrovesical: une tumeur très rare à propos d'un cas

**DOI:** 10.11604/pamj.2016.23.79.9020

**Published:** 2016-03-10

**Authors:** Ali Beddouche, Othemane Fahsi, Adil Kallat, Hicham El Bote, Idriss Ziani, Hachem El Sayegh, Ali Iken, Lounis Benslimane, Yassine Nouini

**Affiliations:** 1 Service d'Urologie A, Hôpital Ibn Sina, Chu Rabat, Maroc

**Keywords:** Schwannoma, retrovesical, benign, surgery, Schwannoma, retrovesical, benign, surgery

## Abstract

Le schwannome est une tumeur le plus souvent bénigne, d'origine nerveuse se développant à partir des cellules de la gaine de Schwann. C'est une tumeur très rare tant par sa fréquence que par sa localisation rétrovésicale. L'examen anatomopathologique et immunohistochimique confirme le type histologique ainsi que le caractère bénin ou malin du schwannome. En raison des récidives, et du risque de transformation maligne, l'exérèse doit être complète. Nous rapportons le cas d'un patient âgé de 39 ans, admis pour une douleur pelvienne chronique à type de pesanteur, des signes irritatifs du bas appareil urinaire, et des troubles de transit. L'imagerie (échographie, TDM, IRM) a révélé la présence d'une masse pelvienne rétrovésicale, à paroi fine, latéralisée à gauche, mesurant 68x70x70 mm, exerçant un effet de masse sur la vessie et le sigmoïde. L'intervention chirurgicale menée par une laparotomie médiane a permis l'exérèse d'une masse retrovésicale bien encapsulée. L'examen anatomopathologique et immunohistochimique ont conclu à un schwannome bénin. La récidive et la transformation maligne bien que rare après chirurgie impose une surveillance post opératoire clinique et tomodensitométrique annuelle.

## Abstract (en)

Schwannoma is usually a benign tumor of nervous origin arising from Schwann sheath cells. It is an extremely rare tumour because of its low incidence and its retrovescical localization. Histological and immunohistochemical examinations confirm the histological type as well as the benign or malignant origin of schwannoma. Because of the risk of recurrence and malignant transformation, excision must be complete. We report the case of a 39 years old patient, hospitalized for chronic pelvic pain accompanied by a sensation of heaviness, lower urinary tract irritation and urinary hesitancy. Diagnostic imaging (ultrasound, CT scan, MRI) of the pelvis showed a left-lateralized retrovescical mass with a thin wall, measuring 68x70x70 mm exerting mass effect on the bladder and the sigmoid. The surgical procedure, a midline laparotomy, allowed the removal of a well encapsulated retrovesical mass. Histological and immunohistochemical examinations concluded to a benign schwannoma. Recidivism and malignant transformation, although rare after surgery, impose postoperative clinical monitoring and annual CT scanning

## Introduction

Le schwannome est une tumeur le plus souvent bénigne, d'origine nerveuse se développant à partir des cellules de la gaine de Schwann. Il peut être uni ou multifocale (maladie de Von Recklinghausen) pouvant siéger dans n'importe quelle partie du corps. Sa localisation retropéritonéale est très rare, et l'est beaucoup plus à l’étage pelvien. La rareté de cette localisation nous incite à rapporter cette observation.

## Patient et observation

**Figure 1:**
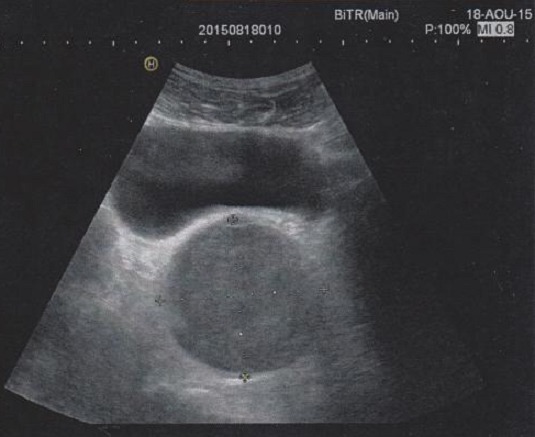
Echographie pelvienne

**Figure 2:**
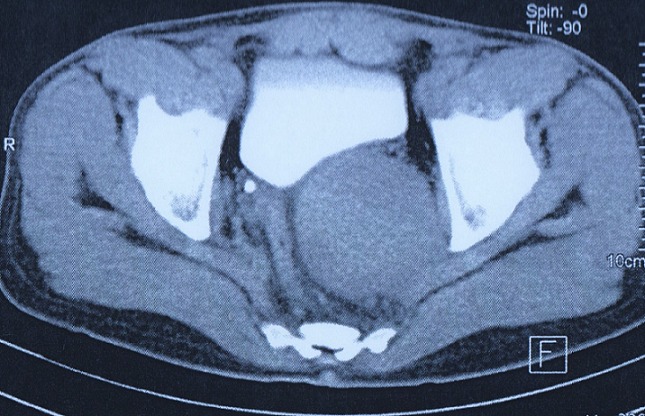
TDM pelvienne

**Figure 3:**
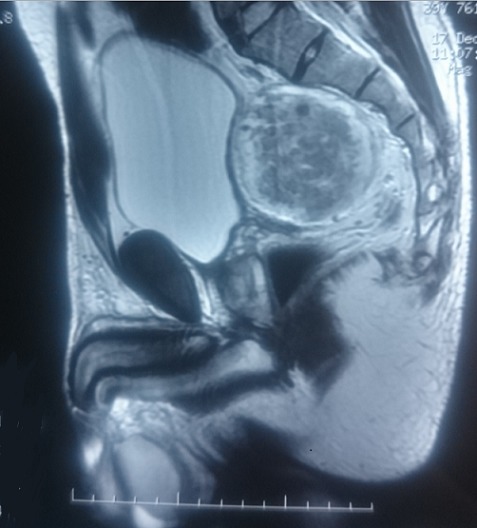
L'IRM pelvienne montre une masse pelvienne rehaussée de façon hétérogène après injection

**Figure 4:**
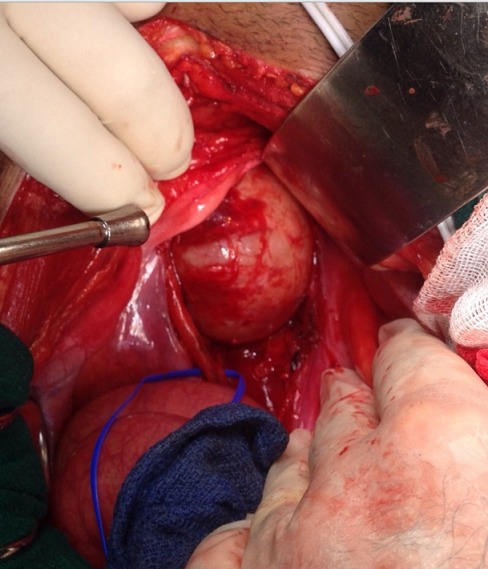
Masse retrovésicale bien encapsulée

**Figure 5:**
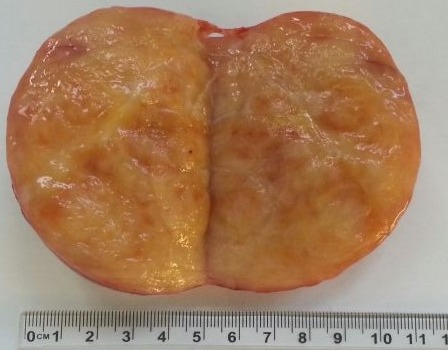
Tumeur nodulaire, encapsulée, de 125g, mesurant 9x6x5 cm, et présentant des zones kystiques

**Figure 6:**
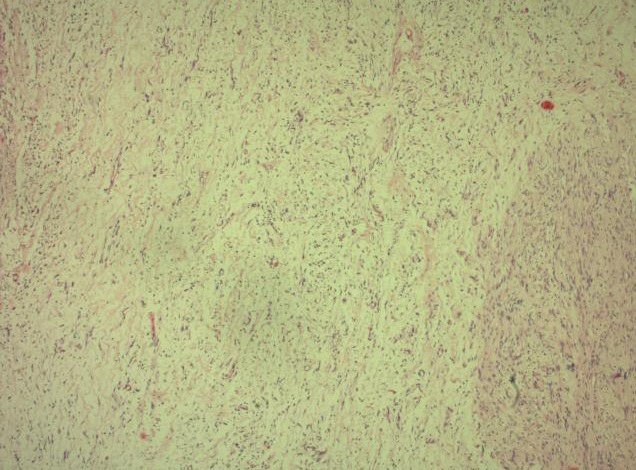
HEx4 prolifération tumorale bénigne formée de 2 secteurs de densité cellulaire variable

**Figure 7:**
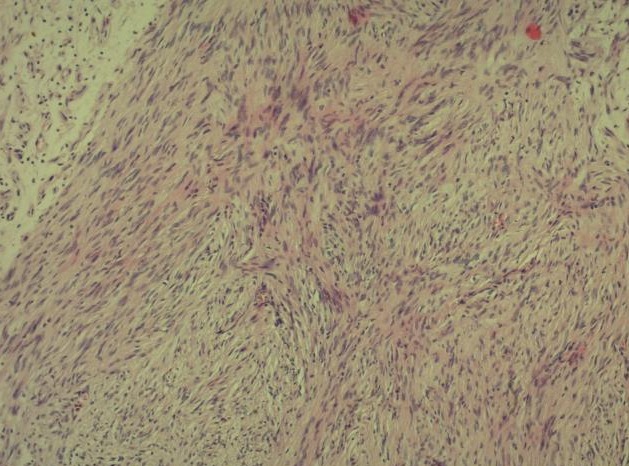
HEx10 prolifération tumorale à cellules fusiformes montrant des palissades nucléaires caractéristiques

Nous rapportons le cas d'un patient âgé de 39 ans, sans antécédent pathologique particulier, présentant une douleur pelvienne chronique à type de pesanteur apparue 6 mois auparavant, accompagnée de signes irritatifs du bas appareil urinaire et de troubles de transit, le tout évoluait dans un contexte d'apyrexie et de conservation de l’état général. L'examen clinique trouvait un patient en bon état général, des constantes hémodynamiques correctes, une légère sensibilité hypogastrique, et un toucher rectal normal. L’échographie pelvienne avait révélé une formation tissulaire hypoéchogène peu homogène à développement rétrovésical sus prostatique mesurant 6 cm (Figure 1). La TDM abdomino-pelvienne avait révélé la présence en rétrovésical d'une masse de 77x74 mm, à paroi fine, de densité semi-liquide, non modifiée après injection, refoulant la paroi postérieure de la vessie (Figure 2). L'IRM avait révélé la présence d'une masse pelvienne latéralisée à gauche arrondie de contours réguliers, en hypo-signal T1 hyper-signal T2, rehaussée de façon hétérogène après injection, mesurant 68x70x70 mm. Elle exerce un effet de masse sur la vessie et le sigmoïde sans signe d'envahissement, elle infiltre focalement les muscles obturateurs interne et externe, refoule les vaisseaux iliaques externes avec disparition du liseré graisseux, et vient au contact de S2 et de l'aile iliaque sans signes de lyse, en bas elle garde un liseré graisseux avec la prostate (Figure 3). L'intervention chirurgicale menée par une laparotomie médiane a permis l'exérèse d'une masse retrovésicale bien encapsulée après repérage de l'uretère gauche, et ligature de deux pédicules fins (Figure 4, Figure 5). Les suites opératoires ont été simples. L'examen anatomo-pathologique (Figure 6, Figure 7) et immunohistochimique (marquage positif à l'anticorps anti PS100) ont conclu à un schwannome remanié sans caractère de malignité.

## Discussion

Le schwannome est une tumeur solide qui se développe à partir des cellules de la gaine nerveuse de Schwann [1, 2]. En 1910, Verocay a décrit la première observation [3, 4]. On doit le terme de schwannome à Masson en 1932 [1, 3]. Souvent bénin, pouvant se présenter sous deux formes: solitaire ou multiple dans le cadre d'une neurofibromatose type2. Le schwannome est une lésion ubiquitaire pouvant siéger dans n'importe quelle partie du corps, membres: 53,1% des cas, tronc: 13%, tête et cou: 13,9% [5]. La localisation retropéritonéale représente 3% des schwannomes et 4% des tumeurs rétropéritonéales primitives. Cette localisation rétropéritonéale représente seulement 0,7% des schwannomes bénins et 1,7% des schwannomes malins [6, 7]. A l’étage pelvien le schwannome est tout aussi rare, les publications n’étant faites que de cas isolés, la tumeur peux intéresser tous les organes du petit bassin y compris les organes génitaux externes [8], sa fréquence est identique dans les deux sexes avec un âge moyen de 51 ans, et des extrêmes allant de 27 à 70 ans [9]. Dans la très grande majorité des cas, les schwannomes pelviens se développent en arrière, dans la concavité sacrée et ont pour origine une racine sacrée ou le plexus hypogastrique. Lorsque la tumeur a un point de départ latéral, elle provient du nerf sciatique, fémoral, ou obturateur [8, 10]. Les schwannomes pelviens se révèlent le plus souvent par un effet de masse sur les organes de voisinage entraînant une pesanteur abdominale, des troubles digestifs (constipation, ténesmes…), ou des troubles urinaires (dysurie, rétention aiguë…). Quand le nerf sciatique ou obturateur sont atteints, on peut avoir une irradiation sensitive dans le membre inférieur. L’échographie abdominopelvienne renseigne sur la nature kystique et solide du schwannome, détermine sa taille, ses rapports et son siège. La TDM abdominopelvienne montre une tumeur solide, siège de calcifications, comportant une composante kystique, bien limitée par une capsule et précise ses rapports et son siège. L'IRM fournit les mêmes renseignements, elle montre une tumeur bien encapsulée avec un hyposignal en T1 et un hypersignal hétérogène en T2. Dans certains cas l'artériographie peux s'avérer utile en préopératoire [10]. Le diagnostic de schwannome est évoqué en préopératoire dans un tiers des cas [10]. La biopsie percutanée est déconseillée par la plupart des auteurs en raison des difficultés d'interprétation, du risque de dissémination néoplasique en cas de tumeur maligne et de l'hyper-vascularisation péritumorale [1, 2]. L'extirpation de ces grosses masses bien que difficile est le plus souvent possible vu le caractère encapsulé de la tumeur dont le siège et le volume déterminent la voie d'abord. En raison des récidives, du risque de transformation maligne, l'exérèse doit être complète [1]. Sur le plan anatomopathologique, le schwannome est caractérisé par la présence de nodules de Verocay, deux contingents sont décrits (les types A et B d'Antoni) [1, 3, 10]. • type A: cellules fusiformes, disposées en faisceaux. Le noyau est ovale. Le cytoplasme est peu abondant. • type B: l'arrangement des cellules fusiformes est hasardeux. Les cellules sont séparées par une matrice colorée de façon hétérogène avec l'hématoxyline éosine et le bleu alcian. La surveillance ultérieure comprend un examen clinique et une TDM réalisées à 6 et 12 mois après l'intervention, puis tous les ans pendant 5 ans [10]. La dégénérescence maligne est exceptionnelle [3, 10].

## Conclusion

Le schwannome bénin retrovésical est une tumeur rare dont le diagnostic est souvent tardif. Les examens paracliniques précisent l'origine retrovésicale, et les rapports aux organes de voisinage. Le traitement de référence est l'exérèse chirurgicale complète. L'examen anatomopathologique et immunohistochimique confirment le type histologique ainsi que le caractère bénin ou malin du schwannome. La récidive et la transformation maligne bien que rare après chirurgie impose une surveillance post opératoire clinique et tomodensitométrique annuelle.
